# Modulation of neurological pathways by *Salvia officinalis* and its dependence on manufacturing process and plant parts used

**DOI:** 10.1186/s12906-019-2549-x

**Published:** 2019-06-13

**Authors:** Carsten Tober, Roland Schoop

**Affiliations:** 1 Rent-a-lab, Aspenhaustr. 25, 72770 Reutlingen, Germany; 2 A.Vogel AG, Grünaustrasse, 9325 Roggwil, Switzerland

**Keywords:** *Salvia officinalis*, Hot flushes, Menopause, Adrenergic, Serotonergic, Muscarinic, μ-Opioid neurotransmitter

## Abstract

**Background:**

*Salvia officinalis* has been used successfully for the treatment of hot flushes and excessive sweating during menopause. However, modes of actions have not been elucidated conclusively. We explored its pharmacology beyond any hormonal activity with a focus on neurologic impulse transmission.

**Methods:**

A hydroalcoholic, thujone-free extract from freshly harvested *Salvia officinalis* leaves (A.Vogel Menosan®) was investigated in an acetylcholinesterase enzyme assay and several receptor binding assays (adrenergic alpha 2A, GABA (benzodiazepine site), GABA_B_; muscarinic M3, μ-opioid, serotonin 5-HT_1A_, serotonin 5-HT_2B_, serotonin 5-HT_2C_ and serotonin transporter). The influence of the manufacturing process on additional extracts from different fresh or dry plant parts was studied.

**Results:**

The *Salvia officinalis* extract replaced 50% of specific ligand binding to GABA_A_ and GABA_B_ receptors at an inhibitory concentration (IC_50_) of 89 and 229 μg/ml, respectively. Strong binding affinity was observed for the adrenergic α_2A_ receptor, μ-opioid receptors, muscarinic M3 receptors, and serotonin 5-HT_1A_ receptors, with IC_50_ values of 15 μg/ml, 20 μg/ml, 25 μg/ml and 19 μg/ml, respectively. Moderate interference with 5-HT_2B_, 5-HT_2C_ receptors, and the human serotonin transporter was found, all with IC_50_ values above 32 μg/ml. Receptor binding data of Salvia extract were confirmed in native female hypothalamic tissue from two women (51 and 37 years old).

Use of freshly harvested Salvia leaves resulted in 2- to 4-fold higher activity/lower IC_50_ values compared to extracts from dried plants or stipes.

**Conclusion:**

Our results suggest potent modulation of neuro-receptors and of serotonin transporters as mode of action for *Salvia officinalis* alcoholic extract, which may normalize thermoregulation and possibly also mental impairment during menopause.

## Background

Hot flushes are the most often mentioned and pivotal symptoms of the menopausal syndrome and are defined as a sudden feeling of heat or burning that starts in the head, neck, upper chest, or back. Sometimes hot flushes are accompanied by red, blotchy skin or palpitation [1]. In fact, an increase of core body temperature has been observed, preceded by chilliness as hot flushes subside [2, 3]. They appear to evolve from disturbed thermoregulation in the hypothalamic region, which is equipped with thermosensitive neurons [4]. Symptoms like sleep disturbance, emotional lability and even depression further add to the syndrome and a high proportion of women are urged to seek medical help [5].

The etiology of hot flushes is unknown, although several mechanisms have been discussed. The reduction of hot flushes with estrogen replacement therapy suggests a hormonal etiology. However, blood levels of estrogens do not appear to correlate with hot flushes [6]. It seems more likely that the decreased estrogen concentrations trigger fundamental alterations within the thermoregulatory system via the hypothalamus and/or in the periphery. The serotonergic and noradrenergic systems are believed to play an immediate role in the maintenance of temperature regulation in the brain, as well as in the periphery. There is supporting evidence that estrogens regulate these two systems by modulating the production, release, and reuptake of serotonin and noradrenaline and the activity of their receptors. Therefore, drugs that are able to restore the altered levels of serotonin and/or noradrenaline caused by estrogen withdrawal are believed to be effective in the treatment of hot flushes.

Therapy of menopausal symptoms increasingly abstains from classical hormone replacement therapy (HRT), taking into account the reported side effects, like breast cancer or heart disease [7]. Although the risk seems to depend on hormonal preparation, composition, and application, as well as on patient characteristics, a basic suspicion remains and novel, tolerable alternatives are sought. Clinical studies have tested selective serotonin reuptake inhibitors (SSRIs) [8], as well as anticholinergic drugs (muscarinic receptor antagonist, oxybutynin) and antihypertonica (α2-receptor agonist like clonidine) with encouraging results; however, indications have so far not been granted by regulatory agencies [9–12].

Firstly, from a pharmacological point of view the investigation of neurotropic substances makes a lot of sense because cholinergic and adrenergic nerve terminals are adjacent to sweat glands [13, 14]. Secondly, norepinephrine, dopamine, 5-hydroxytryptamine (serotonin), and acetylcholine are involved in thermoregulation in the hypothalamus [15, 16]. Whether and where (centrally or at the periphery) a substance will be active finally depends on the ability to cross the blood-brain barrier.

Considering the multifactorial pathological process behind menopausal complaints, multicomponent herbal preparations could present an interesting therapy option. *Salvia officinalis* and *Cimicifuga racemosa* as well as phytoestrogens (e.g., from soya), have been used successfully for decades to treat menopausal complaints. *Salvia officinalis* has traditionally been used against excessive sweating and hot flushes in menopause, to improve lipid status and liver function and for increase of mental capacity as referenced in [17]. In clinical trials a hydroethanolic, thujone-free extract (A.Vogel Menosan®) reduced hot flush intensity and frequency by 64% after 2 months [18, 19]. A Salvia ethanolic extract has been administered over 2 months to decrease symptoms associated with premenstrual syndrome in women, 18–35 years of age [20]. No conclusive pharmacodynamic mode of action has been identified so far. Rahte observed no effects on serotonin re-uptake, very limited acetylcholinesterase (AChE) inhibition, and estrogenic activity in only one subfraction, which was lost in the total extract [21]. Others, however, have also seen AChE inhibition of *Salvia* phenolic mono- and di-terpenes in mice [22, 23], suggesting beneficial effects in Alzheimer’s disease and for cognitive functions [24]. Overall, results are contradictory and seem largely to depend on the manufacturing processes and/or plant species and plant parts used. Biologically active substances in Salvia comprise mono-, di- and triterpenes like 1,8–cineol, carnosic acid, carnosol or ursolic acid as well as phenolic compounds including caffeic- or rosmarinic acid or flavonoids (e.g. quercetin) [25]. Finally, essential oils are considered to contribute to the pharmacological spectrum of the plant [26].

In our experiments, we aimed to screen the actions of a proprietary *Salvia officinalis* extract (A.Vogel Menosan®) on elements of neurologic impulse transmission and to identify influences of the production process on these actions.

## Methods

A thujone-free sage spissum extract (A. Vogel Menosan®, batch 041941) and the originating ethanolic tincture (67% EtOH V/V, batch 040116) were prepared from freshly harvested outer sage leaves (*Salvia officinalis* Folium rec.) at a drug extractant ratio (DER) 1:17. Dry mass content of the spissum extract was 19.2% [m/m] and total (α and β) thujone was < 20 ppm. The plant material was sourced from organic cultivations in Switzerland, verified and manufactured by A.Vogel AG (Roggwil, Switzerland). A.Vogel AG also provided tinctures from fresh plants [FE 150917 (only leaves), FE 150918 (only stipes), and FE 150916 (leaves and stipes)] and from dried plants [FE 150914 (only leaves), FE 150915 (only stipes), and FE 1509163 (leaves and stipes)], which have been produced according to the same extraction procedures as stated above. Dilutions of herbal preparations were made in H_2_O_Milli-Q_. Vehicle control was 67% ethanol for *Salvia officinalis.* Folium rec.T.1:17 and 3.7% saccharose laurate for spissum.

The following material were used for receptor binding analysis: 1-[N-methyl-^3^H] scopolamine methyl chloride ([^3^H]-NMS, hM3) was from GE Healthcare. [^3^H]CGP 54626 (GABA_B_) was purchased from BIOTREND Chemikalien GmbH (Cologne, Germany) and stored under the recommended conditions at − 20 °C. [N-methyl-^3^H]-Ro-15-1788 (GABA_A_-Bz-site), [^3^H]-DAMGO (μ-opioid), [^3^H(G)]-MK-912 (alpha 2A), and [^3^H]-imipramine (hydrochloride, [benzene ring – ^3^H(N)]-, 5-HTT) were from PerkinElmer (Rodgau, Germany). [^3^H]-8-OH-DPAT ([propyl-2,3-ring-1,2,3-^3^H], 5-HT_1A_) and [N-methyl-^3^H]-mesulergine (5-HT_2B_) were from ARC Inc. (St. Louis, USA).

Recombinant human AChE was from Bio-Techne GmbH (Wiesbaden-Nordenstadt, Germany). Human adrenergic α_2A_ receptors were from Merck Millipore. Human serotonin 5-HT_1A_ receptors, human serotonin 5-HT_2C(e)_ receptors, human muscarinic M_3_ receptors, human μ opioid receptors, and human serotonin transporters were from PerkinElmer (Rodgau, Germany). Human serotonin 5-HT_2B_ receptors were from rent-a-lab (Reutlingen, Germany).

Diazepam-ratiopharm® (GABA_A_-Bz-site, non specific) was from ratiopharm GmbH (Ulm, Germany). Baclofen (GABA_B_, non specific), 4-Diphenylacetoxy-N-methylpiperidine methiodide (4-DAMP, M3, non specifc and reference compound), D-Ala^2^,N-Me-Phe^4^,Gly^5^-ol)-ENKEPHALIN (DAGO, DAMGO, μ-opioid, reference compound), Naloxone-HCl (μ-opioid, non specific), 5-Hydroxytryptamine hydrochloride (Serotonin, 5-HT_1A_, non specific), WAY 161503 hydrochloride (5-HT_2C_, reference compound), Imipramine hydrochloride (5-HTT, non specifc and reference compound), acetylthiocholine, 5,5′-Dithiobis(2-nitrobenzoic acid) (DTNB) and neostigmine (reference compound: AChE) were from Sigma (Taufkirchen, Germany). Mianserin hydrochloride (5-HT_2C_, 5-HT_2B_, non specific and reference compound 5-HT_2B_), SR 95531 hydrobromide (GABA_A_-Bz-site, reference compound), CGP 54626 hydrochloride (GABA_B_, reference compound), (±)-8-Hydroxy-2-dipropylaminotetralin hydrobromide (8-OH-DPAT, 5-HT_1A_, reference compound), and Rauwolscine hydrochloride (alpha 2A, non specific and reference compound) were from BIOTREND Chemikalien GmbH (Cologne, Germany).

Human frozen hypothalami were from a healthy 51-year old and a healthy 37-year-old woman (Tissue Solutions Ltd., Glasgow, UK). The tissue was minced in 20 ml 10 mM HEPES pH 7.4, 1 mM EDTA on ice with a disperger (20 s at 28 Trpm) and centrifuged for 15 min at 31,600×g at 4 °C. The pellet was homogenized (10 strokes with a glass-teflon homogenizer, 2000 rounds per minute) at 4 °C and again centrifuged. The latter procedure was repeated once and the pellet finally resupended in 50 mM HEPES pH 7.4, 4 mM MgCl_2_, and 1 mM EDTA; frozen in liquid nitrogen; and stored at − 80 °C until usage.

### Acetylcholinesterase enzyme assay

Inhibition of AChE was assessed by a modified version of the colorimetric method of Ellman et al. [27]. AChE and effector/inhibitor were added to 100 mM phosphate buffer (pH 7.5) and 0.05% Brij L23, and the reaction was started by the addition of acetylthiocholine and DTNB. The thiocholine formed during hydrolysis of acetylthiocholine rapidly reacts with DTNB and releases a yellow 5-thio-2-nitrobenzoic acid anion. The production of this coloured anion was read after 30 min incubation at room temperature by an absorbance microplate reader (Sunrise, Tecan Deutschland GmbH, Crailsheim, Germany) at 405 nm.

The AChE enzyme assay was validated by determination of the IC_50_ value of the prototypic AChE inhibitor neostigmine.

### Receptor binding assays

The GABA_A_-benzodiazepine site binding assay was performed as described by Mehta and Shank [28], with minor modifications. Membranes were incubated for 30 min at 22 °C in 50 mM Tris-HCl (pH 7.4) and 100 mM NaCl with 1 nM [^3^H]-Ro-15-1788. Non-specific binding was determined with 10 μM diazepam.

The GABA_B_ receptor binding assay was performed as described by Asay & Boyd [29] with minor modifications: On the day of the assay, membranes were thawed at room temperature and kept on ice. Membranes were then washed 3 times with 25 volumes of ice-cold assay buffer by centrifugation at 18,000×g for 15 min at 4 °C. The pellet was resuspended and incubated for 90 min at room temperature in 20 mM Tris-HCl (pH 7.4), 120 mM NaCl, 6 mM Glucose, 4.7 mM KCl, 1.8 mM CaCl_2_, 1.2 mM MgSO_4_ and 1.2 mM KH_2_PO_4_ with 2 nM [^3^H]-CGP54626. Non specific binding was determined with 10 μM baclofen.

The alpha_2A_ receptor binding assay was performed according to the data sheet provided by the supplier of the receptor preparation, with modifications. The receptor preparation was incubated for 60 min at 30 °C in 50 mM HEPES (pH 7.4) and 5 mM MgCl_2_ with 1 nM [^3^H]-MK-912. Non specific binding was determined with 3.16 μM rauwolscine.

The serotonin 5-HT_1A_ receptor binding assay was performed according to the data sheet provided by the supplier of the receptor preparation, with modifications. The receptor preparation was incubated for 120 min at 37 °C in 50 mM Tris-H_2_SO_4_ (pH 7.4) and 5 mM MgSO_4_ with 0.5 nM [^3^H]-8-OH-DPAT in the dark. Non specific binding was determined with 10 μM serotonin.

The serotonin 5-HT_2B_ receptor binding assay was performed as described by Wainscott et al. [30] with minor modifications. The receptor preparation was incubated for 120 min at room temperature in 50 mM Tris-HCl (pH 7.4), 0.1% ascorbic acid and 10 μM pargyline with 2 nM [N^6^-methyl-^3^H]-mesulergine. Non-specific binding was determined with 10 μM mianserin.

The serotonin 5-HT_2C(e)_ receptor binding assay was performed according to the data sheet provided by the supplier of the receptor preparation, with modifications. The receptor preparation was incubated for 120 min at 37 °C in 50 mM Tris-HCl (pH 7.4), 0.1% ascorbic acid and 10 μM pargyline with 0.5 nM [N^6^-methyl-^3^H]-mesulergine in the dark. Non-specific binding was determined with 1 μM mianserin.

The μ-opioid receptor binding assay was performed according to the data sheet provided by the supplier of the receptor preparation, with modifications. The receptor preparation was incubated for 60 min at 27 °C in 50 mM Tris-HCl (pH 7.4) and 5 mM MgCl_2_ with 0.6 nM [^3^H]-DAMGO. Non specific binding was determined with 10 μM naloxone.

The muscarinic M3 receptor binding assay was performed according to the data sheet provided by the supplier of the receptor preparation, with modifications. The receptor preparation was incubated for 90 min at 25 °C in 50 mM Tris-HCl (pH 7.4), 2.5 mM MgCl_2_, and 1 mM EDTA with 0.3 nM [^3^H]-NMS. Non specific binding was determined with 1 μM 4-DAMP.

The 5-HTT serotonin transporter binding assay was performed according to the data sheet provided by the supplier of the receptor preparation, with modifications. The receptor preparation was incubated for 30 min at 27 °C in 50 mM Tris-HCl (pH 7.4), 120 mM NaCl and 5 mM KCl with 1.5 nM [^3^H]-imipramine. Non-specific binding was determined with 10 μM imipramine.

All assays were terminated by transfer of the samples onto filter plates (PerkinElmer or Whatman), presoaked in assaybuffer (GABA_A_, GABA_B_, alpha2A and muscarinic M3), treated with 0.5% BSA (μ-opioid), presoaked with 0.5% polyethyleneimine (5-HT_2B_, 5-HT_2C(e)_), or presoaked with 0.1% polyethyleneimine (5-HT_1A_, 5-HTT). Filters were washed four times with 200 μl of ice-cold 50 mM Tris-HCl pH 7.4 (GABA_A_, GABA_B_, Alpha2A, 5-HT_1A_, 5-HT_2B_ 5-HT_2C(e)_, μ-opioid, and muscarinic M3) or 50 mM Tris-HCl pH 7.4/0.5 M NaCl (5-HTT), and filter-bound radioactivity was determined by a microplate reader (Microbeta, Wallac, Finnland).

From every data point non-specific binding was subtracted and data normalized (100% specific binding represents the specific binding of the radioligand to the binding site of the receptor in the absence of any effector).

The IC_50_ value (concentration causing half-maximal inhibition of specific binding) was determined by non-linear regression analysis of the competition curves using the “sigmoidal dose-response” algorithm (GraphPad Prism, San Diego, USA).

Every receptor binding assay was validated by suitable well-characterised reference compounds for the respective receptor. An assay was considered validated if obtained IC_50_ values for the reference compound were within ±0.5 log-units of published data and/or historical data from our laboratory.

## Results

### Acetylcholinesterase

Inhibition of AChE was assessed by a modified version of the colorimetric method of Ellmann et al. [27]. The enzyme assay was validated by the determination of the IC_50_ value of the prototypic AChE inhibitor neostigmine. The obtained IC_50_ value of 16 nM is in good agreement with, for example, Kishibayashi et al. [31], who measured an IC_50_ value of 36 nM for the inhibition of AChE by neostigmine.

Salvia spissum extract inhibited the AChE activity with an IC_50_ value of above 400 μg/ml; a concentration of 400 μg dry mass/ml led to only 33.5% ± 1.1% inhibition (data not shown).

### Adrenergic system

**Fig. 1:**
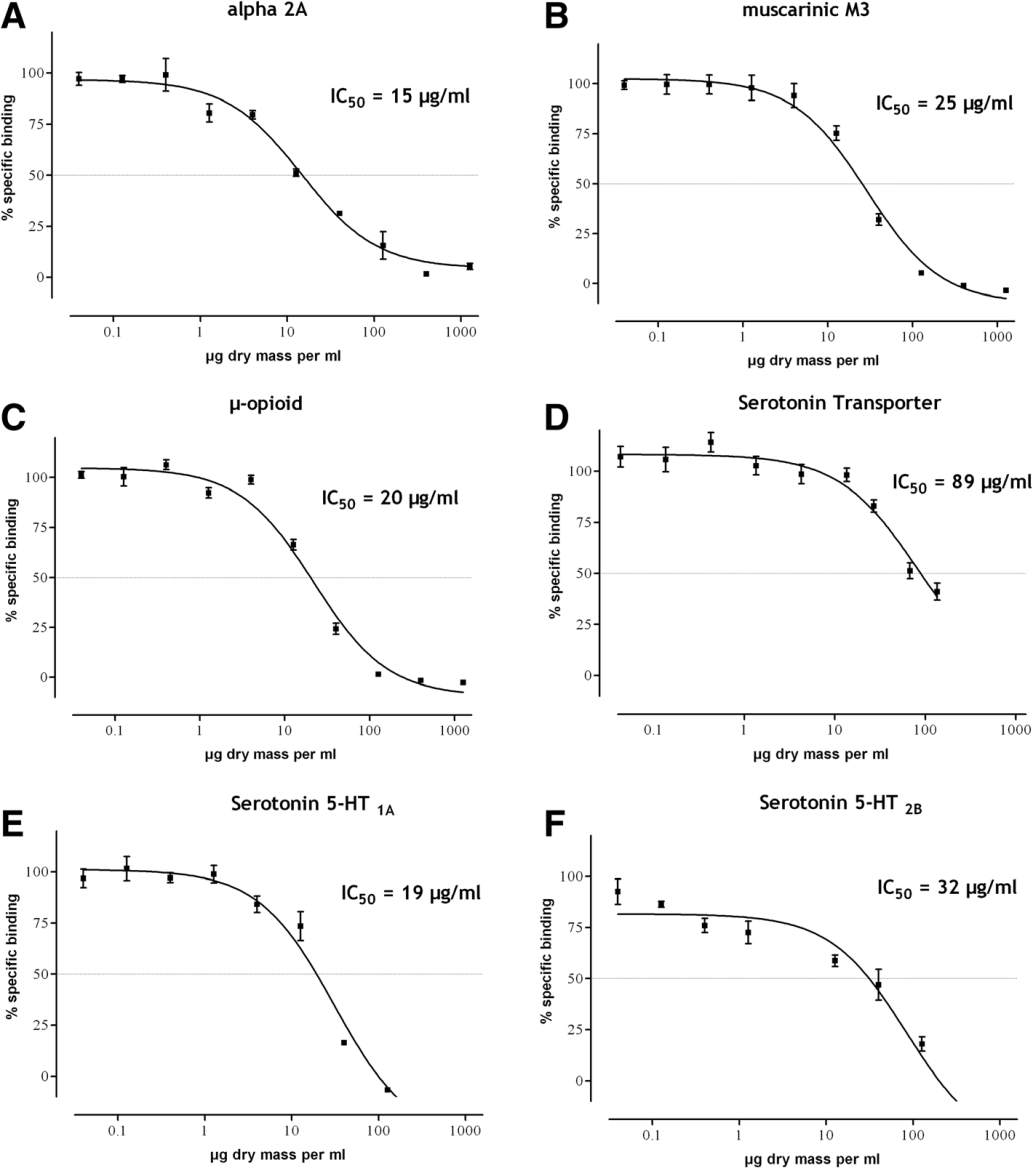
Competition binding at several receptors**.** Competition binding of *Salvia officinalis* extract at the human adrenergic alpha 2A receptor expressed in Chem-1 cells (**a**), the muscarinic M3 receptor expressed in CHO cells (**b**), the human μ-opioid receptor expressed in CHO-K1 cells (**c**), the human serotonin transporter expressed in HEK293 cells (**d**, Salvia off. tincture), the human serotonin 5-HT_1A_ receptor expressed in HEK293-EBNA cells (**e**), and the human serotonin 5-HT_2B_ receptor expressed in CHO-K1 cells (**f**). Summary curves over 2–3 independent experiments performed in duplicate (mean specific binding ± S.E.M.). The calculated IC_50_ values are indicated above the figure. The horizontal dashed line marks 50% specific binding

The α_2A_ adrenoceptor antagonist rauwolscine competed for the binding of [^3^H]-MK-912 to Chem-1 cells expressing the human adrenergic α_2A_ receptor in a concentration-dependent manner with an IC_50_ value of 8.2 nM. *Salvia officinalis* extract competed with the binding of the receptor-specific radioligand of the human adrenergic α_2A_ receptor at a low IC_50_ value of 15 μg/ml (Fig. 1a, Table 1), while the vehicle control (saccharose laureate) yielded an IC_50_ value corresponding to more than 1000 μg dry mass/ml (data not shown).

**Table 1:** Summary of results

receptor or Transporter	IC_50_, μg dry mass/ml
Adrenergic alpha 2A	15 (8.7–24.5)
GABA (Bz-site)	89 (64–124)
GABA_B_	229 (119–440)
Muscarinic M3	25 (19–72)
μ-opioid	20 (12–35)
5-HTT	89 (28–283)
Serotonin 5-HT_1A_	19 (12–32)
Serotonin 5-HT_2B_	32 (14–71)
Serotonin 5-HT_2C_	60 (25–146)
Enzyme	
Acetylcholinesterase	> 400

Data table to Fig. 1 supplemented by competition binding of *Salvia officinalis* extract at the GABA_A_ (Bz-site) receptor, the GABA_B_ receptor, the human serotonin 5-HT_2B_ receptor expressed in CHO-K1 cells, and inhibition of acetylcholinesterase. Data are IC_50_ values and 95% confidence intervals of summary curves over 2–3 independent experiments performed in duplicate

In membranes from native female hypothalami the reference antagonist rauwolscine and the test item, *Salvia officinalis* extract, competed for the binding of [^3^H]-MK-912 with IC_50_ values of 3.2 nM and 224 μg/ml, respectively (data not shown).

### Muscarinic M3 receptor

The specific muscarinic M3 receptor antagonist 4-DAMP competed for the binding of [^3^H]-NMS to membranes of CHO cells expressing the human muscarinic M3 receptor in a concentration-dependent manner (IC_50_ = 2.1 nM, data not shown). *Salvia officinalis* extract competed with the binding of the receptor-specific radioligand of the human muscarinic M3 receptor at a low IC_50_ value of 25 μg dry mass/ml (Fig. 1b, Table 1), while the vehicle control (saccharose laureate) yielded an IC_50_ value corresponding to about 300 μg dry mass/ml (data not shown).

In membranes from human native female hypothalami, the reference antagonist 4-DAMP and the test item, *Salvia off.* Extract, competed for the binding of [^3^H]-NMS with IC_50_ values of 3.9 nM and 129 μg/ml, respectively (data not shown).

### Opioid system

DAMGO, an enkephalin analog that is a selective agonist at μ-opioid receptors, competed for the binding of [^3^H]-DAMGO to membranes of CHO-K1 cells expressing the human μ-opioid receptor in a concentration-dependent manner. The obtained IC_50_ value of 0.9 nM is in accordance with published data [32]. Salvia extract competed with the radioligand for μ-opioid binding sites with an IC_50_ value of 20 μg/ml (Fig. 1c and Table 1). In membranes from human native female hypothalami, no binding sites for [^3^H]-DAMGO could be detected (data not shown), indicating either the absence of the μ-opioid receptor in the hypothalamus or a degradation of the binding site during the tissue handling procedure. In the former case, the action of the extract would be located in signal transduction rather than in central thermoregulation.

### GABA (γ-aminobutyric acid), GABAergic system

Representative of the GABAergic system, the binding of the *Salvia officinalis* extract to the benzodiazepine site of the GABA_A_ receptor and the GABA_B_ receptor was investigated, and relatively high IC_50_ values of 89 μg/ml and 229 μg/ml, respectively, were obtained (Table 1). The assays were validated by the IC_50_ determination of the reference compounds diazepam (Bz-site of GABA_A_) and CGP-54626 (GABA_B_); IC_50_ values of 39 nM and 4.7 nM, respectively, were obtained (data no shown).

### Serotonergic system

Serotonin reuptake was investigated via a serotonin transporter binding assay. The binding assay delivers comparable data to direct uptake measurements, but is much easier to handle. The tricyclic antidepressant imipramine – a reference inhibitor of serotonin transporters – competed for the binding of [^3^H]-imipramine to human serotonin transporters expressed in HEK293 cells in a concentration-dependent manner (Fig. 1). The calculated IC_50_ value of 2.9 nM is in good agreement with Tatsumi et al. [33], who determined a K_D_ of 1.4 nM for [^3^H]-imipramine binding to human serotonin transporters. Due to interference of the spissium with the assay system, the tincture was investigated and an IC_50_ value of 89 μg/ml was determined for the competition, with [^3^H]-imipramine binding to human serotonin transporters (Fig. 1d, Table 1). In serotonin reuptake experiments with synaptosomes, even lower IC_50_ values of around 30 μg/ml were obtained for the extract (data not shown).

To complete the investigation of the serotonergic system, the binding of the *Salvia officinalis* to one member of the 5-HT_1_-family, namely the 5-HT_1A_-receptor, and to two members of the 5-HT_2_-family, namely the 5-HT_2B_ and the 5-HT_2C(e)_ receptors, was investigated. Binding assays were validated by IC_50_ determinations with specific reference compounds for each respective receptor: 8-OH-DPAT (5-HT_1A_, 0.7 nM), mianserin (5-HT_2B_, 6.5 nM) and WAY 161503 (5-HT_2C(e)_, 163 nM).

*Salvia officinalis* extract competed for binding to the 5-HT_1A_ receptors, 5-HT_2B_ receptors and 5-HT_2C(e)_ receptors with IC_50_ values of 19 μg/ml, 32 μg/ml, and 60 μg/ml, respectively (Fig. 1e and f; Table 1).

In membranes from human native female hypothalami, no specific binding of the radioligand for the 5-HT_1A_, [^3^H]-8-OH-DPAT, could be observed, indicating the absence of the serotonin 5-HT_1A_ receptor at least in the investigated preparation. In contrast, binding of [^3^H]-mesulergine to the native female hypothalami preparation could be displaced by WAY 161503, the reference compound for the serotonin HT_2C_ receptor (IC_50_ = 347 nM). *Salvia officinalis* extract, competed for the binding of [^3^H]-mesulergine, with a low IC_50_ value of 28 μg/ml (data not shown).

### Human native hypothalamic membranes

The initial experiments were mostly performed on human receptors originating from expression systems, (e.g., DNA encoding a human receptor is introduced into CHO cells and overexpressed therein, resulting in a relatively artificial system [34]). Therefore, we decided to confirm the results using membrane preparations from healthy human female hypothalami. In most cases (5-HT_2C_, 5-HTT, and muscarinic M3) the reference compounds displayed 2- to 3-fold lower affinities to the native human receptors than to the human recombinant receptors. Subsequently, the IC_50_ values of *Salvia officinalis* were 2- to 5-fold higher, as well. At the serotonin 5-HT_2C_ receptor an about 1.8-fold lower IC_50_ value was measured in the native human tissue compared to the recombinant receptor preparation. In one case (adrenergic alpha 2A), a 2.6-fold lower IC_50_ value and a 17-fold higher IC_50_ value were measured for the native human tissue compared to the recombinant receptor preparation for the reference compound and the *Salvia officinalis* extract, respectively. In two cases (μ-opioid and serotonin 5-HT_1A_), no specific binding could be observed with the radioligands for the respective receptors, indicating the absence of those receptors at least in the native hypothalamic membrane preparation used. One reason could be the total absence of μ-opioid and serotonin 5-HT_1A_ receptors in human female hypothalamus, indicating a peripheral rather than central role for these receptors in thermoregulation. Another reason could be that the sample treatment, especially the time between sample taking and shock freezing, was too long, which resulted in degradation of these receptors and/or the binding sites or surroundings of these receptors. The time period between sample taking and shock freezing could also be responsible for lower affinity of the reference compound and test item to some receptors.

Altogether, however, data initially gained on human recombinant receptors could be confirmed on native female hypothalamic receptors, underlining the significance of the findings regarding the influence of Salvia extract on central thermoregulation.

### Influence of manufacturing process

**Fig. 2:**
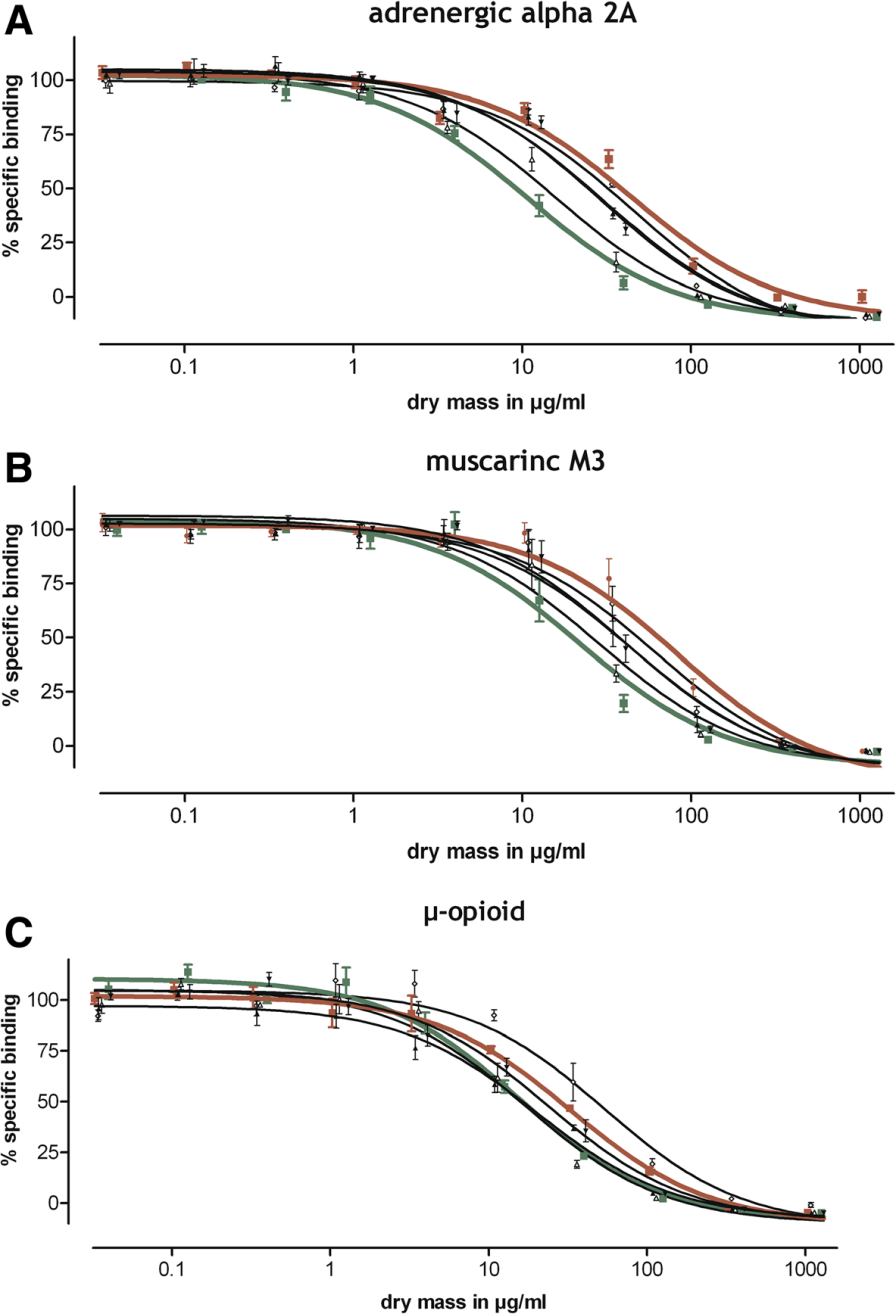
Influence of the manufacturing process. Competition binding of different ethanolic *Salvia officinalis* extracts (highlighted: green of fresh leaves, brown of dry stipes) at the human adrenergic alpha 2A receptor expressed in Chem-1 cells (**a**), the muscarinic M3 receptor expressed in CHO cells (**b**), and the human μ-opioid receptor expressed in CHO-K1 cells (**c**). Summary curves over 2 independent experiments performed in duplicate (mean specific binding ± S.E.M.)

In a second step, we investigated whether the manufacturing process has an influence on receptor binding affinity. In particular, we were interested in whether there is a difference between extracts of dry or fresh material and between the various plant parts used. Freshly-harvested and dried above-ground plant parts were dissected into stipes alone and leaves alone or were left as stipes and leaves. The plant material was subjected to hydroalcoholic (67% v/v) extraction and was then examined for receptor binding potency in the binding assays for the adrenergic alpha_2A_, muscarinic M3, and μ-opioid receptors. An unequivocal pattern of inhibition resulted, with highest binding affinities seen for extracts prepared from freshly harvested *Salvia officinalis* in comparison to samples obtained from dried plants (Fig. 2, Table 2). Superior effects were seen on the level of all three receptors tested. Regarding the plant parts used, leaves yielded superior effects over stipes, while the combination gave an average inhibition.

**Table 2:** Influence of the manufacturing process (Data table to Fig. 2)

alpha 2A		Muscarinic M3		μ-opioid	
Test item	IC_50_, μg dry mass/ml	Test item	IC_50_, μg dry mass/ml	Test item	IC_50_, μg dry mass/ml
**Leaves (fresh)**	**11**	**Leaves (fresh)**	**22**	**Leaves (fresh)**	**16**
Leaves + Stipes (fresh)	16	Leaves + Stipes (fresh)	28	Leaves + Stipes (fresh)	17
Leaves + Stipes (dry)	29	Leaves (dry)	41	Leaves + Stipes (dry)	20
Leaves (dry)	30	Leaves + Stipes (dry)	44	Leaves (dry)	24
*Stipes (dry)*	*45*	Stipes (fresh)	61	*Stipes (dry)*	*33*
Stipes (fresh)	46	*Stipes (dry)*	*83*	Stipes (fresh)	54

Competition binding of different ethanolic *Salvia officinalis* extracts (highlighted in bold and italic: bold of fresh leaves, italic of dry stipes) at the adrenergic alpha 2A receptor, the muscarinic M3 receptor, and the human μ-opioid receptor. Data are the IC_50_ values of summary curves over 2 independent experiments performed in duplicate

For example, alcoholic extracts prepared from freshly harvested *Salvia officinalis* leaves produced lowest IC_50_ values of 11 μg/ml, 22 μg/ml, and 16 μg/ml for the adrenergic alpha_2A_, muscarinic M3, and μ-opioid receptor, respectively. For comparison, the same manufacturing applied to dried *Salvia officinalis* stipes yielded IC_50_ values of 45 μg/ml, 83 μg/ml and 33 μg/ml for the adrenergic alpha_2A_, muscarinic M3, and the μ-opioid receptor, respectively, which were up to 4 times higher (Table 2).

## Discussion

The aim of our investigation was to screen a proprietary extract prepared from freshly harvested *Salvia officinalis* leaves (A.Vogel Menosan®) for a variety of elements involved in neurological impulse transmission to explain in vivo observed benefits on vasomotor and cognitive complaints.

The strongest effects were seen for muscarinic M3, adrenergic α_2A_, and μ-opioid receptors. *Salvia officinalis* competed for the radioligands to the specifc binding sites with IC_50_ values around 20 μg/ml. The observed inhibitory concentrations were much lower than IC_50_ values reported so far. For example, for muscarinic receptors an IC_50_ value of 2000 μg/ml has been reported [35], which is not likely to play a significant physiological role in vivo. All affected receptors play important roles in thermoregulation and menopause pathogenesis at varying locations and nodal points.

Cholinergic and adrenergic nerve terminals are immediately distributed to eccrine sweat glands and muscarinic antagonists scopolamine or hyoscyamine were found to directly inhibit sweating via interaction with M3 [13, 14]. Likewise, adrenergic agonists (clonidine) were able to reduce the number of provoked hot flushes [9] through action on the α_2A_ adrenergic receptor. With its concomitant action on M3 and adrenoceptor alpha2A, we suggest that Salvia components affect sweating directly at the peripheral site of action.

Salvia extract also competed with the radioligand for μ-opioid binding sites at a low IC_50_ value of 20 μg/ml. This finding is interesting in view of μ-opioid ligands (e.g., β-endorphines) playing a central role in thermoregulation [16]. Black cohosh extracts have recently been found to bind to μ-opioid receptors, albeit at considerably higher concentrations of 165.9 μg/ml [36]. Black cohosh has – just like Salvia – successfully been used in menopausal complaints like hot flushes. μ-opioid receptors, together with muscarinic M3 mentioned above and adrenoceptor alpha2, are prominently expressed in the hypothalamus region to control neuronal thermoregulation. Only limited knowledge on blood-barrier passage of *Salvia officinalis* is available, but some (etheric oils and monoterpenes like 1,8-cineol) have the potential to indeed reach the hypothalamic region due to their size and structure [37]. In addition to peripheral effects on gland secretion we thus postulate a central nervous activity of Salvia, which would not only explain effects on vasomotor complaints, but also on cognitive and mood levels.

Versatile but still specific effects were finally seen on the level of the serotonergic system, with potent inhibition primarily of serotonin 5-HT_1A_ receptor rather than on serotonin 5-HT_2B_ or 5-HT_2C_. Serotonin transporters were affected, with IC_50_ values between 30 and 89 μg/ml (uptake into synaptosomes and binding to the transporter, respectively). However, negligible effects of Salvia extract were found on AChE enzyme. Our AChE data are in contrast to data for *S. lavendula* essential oil administered to rats, but in agreement with own in vitro studies to show minimal inhibition of Menosan® at concentrations of 100–200 μg/ml [21]. On the other side, Rahte [21] did not observe any effect on serotonin uptake into recombinant HEK293-cells expressing hSERT receptors for concentrations up to 125 μg/ml. Overall, the combined action on the serotonergic system seems especially interesting for the treatment of hot flushes: an effect primarily on the presynaptic 5-HT_1A_ and influence of re-uptake, and to a lesser extent on postsynaptic 5-HT_2_ receptors.

Negligible effects were finally obtained on GABA_A_ and GABA_B_ receptors (IC_50_ values of 89 and 229 μg/ml for the Salvia extract). Therefore, inhibition of GABA receptors seem to contribute less to the overall pharmacological profile of *Salvia officinalis*.

Overall, our results suggest for the first time a broad pharmacodynamic spectrum for *Salvia officinalis*, with specific effects seen on adrenergic α_2A_, muscarinic M3, and μ-opioid receptors, as well as on serotonin neurotransmission. This is of primary interest, as no significant estrogenic activity of *Salvia off.* Could be shown [21]. Compared to usually high-dosed monodrugs, a wider activity range can be achieved with a combination of active ingredients in one plant extract. This also eliminates side effects due to lower doses of the single compounds in most cases. In addition, phytopharmaca follow the rationale that a complex multifactorial pathophysiology (like in the case of hot flushes) can be managed more effectively through the use of correspondingly composed multidrug mixtures [38]. This concept aligns well with modern treatment options in the therapy of AIDS, cancer, or hypertension, which successfully use 3–5 single synthetic ingredients.

The degree of inhibition (i.e., the affinity to these receptors, as measured by receptor binding) depends crucially on the manufacturing process, as well as on the source of plant material. The highest binding affinities (lowest IC_50_ values) were found for hydroethanolic extracts from fresh plant leaves. Dry plant material and/or supplementing leaves with stipes leads to lower affinities (higher IC_50_ values). Differences apply to all investigated receptors and are on the order of 2- to 4-fold, therefore, extracts from fresh leaves are likely to be more than 2- to 4-fold more active than (for example) extracts from dry stipes. Our results demonstrate a strong bioactivity of *Salvia officinalis* despite depletion of α/β-thujone in the researched product. Further research on thujone-free Salvia species may be warranted to corroborate our findings.

## Conclusions

*Salvia officinalis* influences neurological pathways that are known targets of effective pharmaceuticals utilized in the treatment of menopause and hot flushes. Maximal extract activity is achieved when extracts are manufactured from freshly harvested sage leaves.

## Data Availability

The datasets used and/or analysed during the current study are available from the corresponding author on reasonable request.

## Abbreviations

5-HTT: Serotonin transporter
AChE: Acetylcholinesterase
DTNB: 5,5′-Dithiobis(2-nitrobenzoic acid)
HRT: Hormone replacement therapy
IC_50_: Concentration causing half-maximal inhibition
SSRIs: Selective serotonin reuptake inhibitors

## References

[1] Shanafelt TD, Barton DL, Adjei AA, Loprinzi CL (2002). Pathophysiology and treatment of hot flashes. Mayo Clin Proc.

[2] Freedman RR, Norton D, Woodward S, Cornelissen G (1995). Core body temperature and circadian rhythm of hot flashes in menopausal women. J Clin Endocrinol Metab.

[3] Freedman RR (1998). Biochemical, metabolic, and vascular mechanisms in menopausal hot flashes. Fertil Steril.

[4] Lomax P, Schonbaum E (1993). Postmenopausal hot flushes and their management. Pharmacol Ther.

[5] Hahn PM, Wong J, Reid RL (1998). Menopausal-like hot flashes reported in women of reproductive age. Fertil Steril.

[6] Andrikoula M, Prelevic G (2009). Menopausal hot flushes revisited. Climacteric.

[7] The 2012 Hormone therapy position statement of: the North American Menopause Society. Menopause 2012; 19(3):257–271. 10.1097/gme.0b013e31824b970a PMC3443956 22367731

[8] Carroll DG, Kelley KW (2009). Use of antidepressants for management of hot flashes. Pharmacotherapy.

[9] Freedman RR, Woodward S, Sabharwal SC (1990). Alpha 2-adrenergic mechanism in menopausal hot flushes. Obstet Gynecol.

[10] Joffe H, Guthrie KA, LaCroix AZ, Reed SD, Ensrud KE, Manson JE (2014). Low-dose estradiol and the serotonin-norepinephrine reuptake inhibitor venlafaxine for vasomotor symptoms: a randomized clinical trial. JAMA Intern Med.

[11] Nagamani M, Kelver ME, Smith ER (1987). Treatment of menopausal hot flashes with transdermal administration of clonidine. Am J Obstet Gynecol.

[12] Laguardia KD. Treatment of hot flashes using muscarinic receptor antagonists such as oxybutynin. WO2007143486A2, 2007.

[13] Cheshire WP, Fealey RD (2008). Drug-induced hyperhidrosis and hypohidrosis: incidence, prevention and management. Drug Saf.

[14] Uno H (1977). Sympathetic innervation of the sweat glands and piloarrector muscles of macaques and human beings. J Invest Dermatol.

[15] Sessler DI, Miller RD (2005). Temperature monitoring. Miller’s anesthesia.

[16] Spetz Holm AC, Frisk J, Hammar ML (2012). Acupuncture as treatment of hot flashes and the possible role of calcitonin gene-related peptide. Evid Based Complement Alternat Med.

[17] Jakovljevic M, Jokic S, Molnar M, Jasic M, Babic J, Jukic H, et al. Bioactive profile of various Salvia officinalis L. preparations. Plants (Basel). 2019;8(3). 10.3390/plants8030055 PMC6473381 30845696

[18] Bommer S, Klein P, Suter A (2011). First time proof of sage's tolerability and efficacy in menopausal women with hot flushes. Adv Ther.

[19] Sadeghi AH, Bakhshil M, Behboodi MZ, Goodarzi S, Haghani H (2013). Effect of sage extract on hot flashes in postmenopausal women. Comp Med J Nurs Mid.

[20] Abdnezhad R, Simbar M, Sheikhan Z, Mojab F, Nasiri M (2019). Salvia officinalis reduces the severity of the premenstrual syndrome. Complement Med Res.

[21] Rahte S, Evans R, Eugster PJ, Marcourt L, Wolfender JL, Kortenkamp A (2013). Salvia officinalis for hot flushes: towards determination of mechanism of activity and active principles. Planta Med.

[22] Sallam A, Mira A, Ashour A, Shimizu K (2016). Acetylcholine esterase inhibitors and melanin synthesis inhibitors from Salvia officinalis. Phytomedicine.

[23] Smach MA, Hafsa J, Charfeddine B, Dridi H, Limem K (2015). Effects of sage extract on memory performance in mice and acetylcholinesterase activity. Ann Pharm Fr.

[24] Akhondzadeh S, Noroozian M, Mohammadi M, Ohadinia S, Jamshidi AH, Khani M (2003). Salvia officinalis extract in the treatment of patients with mild to moderate Alzheimer's disease: a double blind, randomized and placebo-controlled trial. J Clin Pharm Ther.

[25] Lu Y, Foo LY (2002). Polyphenolics of Salvia--a review. Phytochemistry.

[26] Fu Z, Wang H, Hu X, Sun Z, Han C (2013). The pharmacological properties of *Salvia* essential oils. J.Appl.Pharm.Sci..

[27] Ellman GL, Courtney KD, Andres V, Featherstone RM (1961). A new and rapid colorimetric determination of acetylcholinesterase activity. Biochem Pharmacol.

[28] Mehta AK, Shank RP (1995). Characterization of a benzodiazepine receptor site with exceptionally high affinity for Ro 15-4513 in the rat CNS. Brain Res.

[29] Asay MJ, Boyd SK (2006). Characterization of the binding of [3H]CGP54626 to GABAB receptors in the male bullfrog (Rana catesbeiana). Brain Res.

[30] Wainscott DB, Cohen ML, Schenck KW, Audia JE, Nissen JS, Baez M (1993). Pharmacological characteristics of the newly cloned rat 5-hydroxytryptamine2F receptor. Mol Pharmacol.

[31] Kishibayashi N, Ishii A, Karasawa A (1994). Inhibitory effects of KW-5092, a novel gastroprokinetic agent, on the activity of acetylcholinesterase in Guinea pig ileum. Jpn J Pharmacol.

[32] Wang JB, Johnson PS, Persico AM, Hawkins AL, Griffin CA, Uhl GR (1994). Human mu opiate receptor. cDNA and genomic clones, pharmacologic characterization and chromosomal assignment. FEBS Lett.

[33] Tatsumi M, Jansen K, Blakely RD, Richelson E (1999). Pharmacological profile of neuroleptics at human monoamine transporters. Eur J Pharmacol.

[34] Felder CC, Schober DA, Tu Y, Quets A, Xiao H, Watt M (2017). Translational pharmacology of the metabotropic glutamate 2 receptor-preferring agonist LY2812223 in the animal and human brain. J Pharmacol Exp Ther.

[35] Wake G, Court J, Pickering A, Lewis R, Wilkins R, Perry E (2000). CNS acetylcholine receptor activity in European medicinal plants traditionally used to improve failing memory. J Ethnopharmacol.

[36] Rhyu MR, Lu J, Webster DE, Fabricant DS, Farnsworth NR, Wang ZJ (2006). Black cohosh (Actaea racemosa, Cimicifuga racemosa) behaves as a mixed competitive ligand and partial agonist at the human mu opiate receptor. J Agric Food Chem.

[37] Moss M, Oliver L (2012). Plasma 1,8-cineole correlates with cognitive performance following exposure to rosemary essential oil aroma. Ther Adv Psychopharmacol.

[38] Wagner H (2006). Multitarget therapy--the future of treatment for more than just functional dyspepsia. Phytomedicine.

